# Genomic Prediction Accuracy for Resistance Against *Piscirickettsia salmonis* in Farmed Rainbow Trout

**DOI:** 10.1534/g3.117.300499

**Published:** 2017-12-18

**Authors:** Grazyella M. Yoshida, Rama Bangera, Roberto Carvalheiro, Katharina Correa, René Figueroa, Jean P. Lhorente, José M. Yáñez

**Affiliations:** * Facultad de Ciencias Veterinarias y Pecuarias, Universidad de Chile, Santiago 8820808, Chile; † Animal Science Department, School of Agricultural and Veterinarian Sciences, São Paulo State University, Campus of Jaboticabal, 14884-900, Brazil; ‡ Akvaforsk Genetics, 6600 Sunndalsora, Norway; § Aquainnovo, Puerto Montt, Chile; ** Núcleo Milenio de Salmónidos Invasores, Concepción, Chile

**Keywords:** disease resistance, genomic selection, *Oncorhynchus mykiss*, reliability, GenPred, Shared Data Resources

## Abstract

Salmonid rickettsial syndrome (SRS), caused by the intracellular bacterium *Piscirickettsia salmonis*, is one of the main diseases affecting rainbow trout (*Oncorhynchus mykiss*) farming. To accelerate genetic progress, genomic selection methods can be used as an effective approach to control the disease. The aims of this study were: (i) to compare the accuracy of estimated breeding values using pedigree-based best linear unbiased prediction (PBLUP) with genomic BLUP (GBLUP), single-step GBLUP (ssGBLUP), Bayes C, and Bayesian Lasso (LASSO); and (ii) to test the accuracy of genomic prediction and PBLUP using different marker densities (0.5, 3, 10, 20, and 27 K) for resistance against *P. salmonis* in rainbow trout. Phenotypes were recorded as number of days to death (DD) and binary survival (BS) from 2416 fish challenged with *P. salmonis*. A total of 1934 fish were genotyped using a 57 K single-nucleotide polymorphism (SNP) array. All genomic prediction methods achieved higher accuracies than PBLUP. The relative increase in accuracy for different genomic models ranged from 28 to 41% for both DD and BS at 27 K SNP. Between different genomic models, the highest relative increase in accuracy was obtained with Bayes C (∼40%), where 3 K SNP was enough to achieve a similar accuracy to that of the 27 K SNP for both traits. For resistance against *P. salmonis* in rainbow trout, we showed that genomic predictions using GBLUP, ssGBLUP, Bayes C, and LASSO can increase accuracy compared with PBLUP. Moreover, it is possible to use relatively low-density SNP panels for genomic prediction without compromising accuracy predictions for resistance against *P. salmonis* in rainbow trout.

In 1989, *Piscirickettsia salmonis* was identified as a pathogenic bacterium causing salmonid rickettsial syndrome (SRS) in farmed coho salmon (*Oncorhynchus kisutch*) in Chile (Branson and Diaz-Munoz 1991; Cvitanich *et al.* 1991). Since then, *P. salmonis* has been confirmed as the causative agent for SRS in coho salmon, Atlantic salmon (*Salmo salar*), and rainbow trout (*Oncorhynchus mykiss*) in several countries, including Norway, Canada, Scotland, Ireland, and Chile (Fryer and Hedrick 2003; Rozas and Enríquez 2014). The economic losses related to SRS in Chile in the year 2012 were US$450 million, owing to mortality, antibiotic treatment, and vaccinations (Camussetti *et al.* 2015)

Currently, treatment for bacterial diseases in the aquaculture industry is predominantly based on antibiotics (Peña *et al.* 2016). Although several vaccines are available for prevention of SRS, none of them provide complete protection against *P. salmonis* in field conditions (Kuzyk *et al.* 2001; Tobar *et al.* 2011). In addition, selective breeding can be used to alleviate disease problems. The levels of genetic variation for resistance to *P. salmonis*, with heritability values ranging from 0.11 to 0.41, have demonstrated the feasibility to improve the trait by means of artificial selection in salmon breeding populations (Yáñez *et al.* 2013, 2014, 2016a; Lhorente *et al.* 2014).

With the recent advances in genotyping methods and the development of single-nucleotide polymorphism (SNP) panels for salmonids (Houston *et al.* 2014; Palti *et al.* 2015; Yáñez *et al.* 2016b; Macqueen *et al.* 2017), genetic markers linked with quantitative trait loci (QTL) can be identified and implemented in breeding programs through marker-assisted selection (MAS) (Yáñez *et al.* 2014). For example, in Atlantic salmon, one major QTL for infectious pancreatic necrosis virus resistance was detected, explaining 29 and 83% of the phenotypic and genetic variances, respectively (Gheyas *et al.* 2010; Houston *et al.* 2010, 2008a,b). This QTL has been successfully used in MAS programs in this species (Moen *et al.* 2015). However, genome-wide association studies (GWAS) in Atlantic salmon suggested that resistance against *P. salmonis* is a trait with moderate polygenic control, with many markers explaining a small proportion of the genetic variance (Correa *et al.* 2015). The complexity of this trait and the absence of QTL with major effects suggest that the implementation of MAS could be not successful in this particular case. By contrast, genomic selection (GS) will be the most appropriate way to incorporate the genomic information and accelerate the genetic progress for traits where the markers have small effects.

Genomic evaluations using dense SNP markers have been shown to increase accuracy of estimated breeding values (EBV) compared with pedigree-based methods for different economically important traits in Atlantic salmon (Ødegård *et al.* 2014; Tsai *et al.* 2015, 2016; Bangera *et al.* 2017; Correa *et al.* 2017; Sae-Lim *et al.* 2017) and rainbow trout (Vallejo *et al.* 2016, 2017). Different GS methods have been tested and prediction accuracy varies depending on the method used, which mainly differ with respect to the assumption about marker effects and the genetic relationship matrix calculation. The genomic best linear unbiased predictor (GBLUP) assumes that all marker effects come from a normal distribution (Meuwissen *et al.* 2001; VanRaden 2008), and the relationship matrix is calculated using genomic information only. The single-step GBLUP (ssGBLUP) assumes the same normal distribution for marker effects; however, it uses a combination of pedigree and genomic information to determine the additive genetic relationship matrix (Aguilar *et al.* 2010). In general, Bayesian methods assume more flexible and nonnormal distributed marker effects. For instance, the Bayes C method assumes that SNP effects have independent and identical mixture distributions (Habier *et al.* 2011), whereas the Bayesian Lasso (LASSO) assumes a double exponential prior distribution for variances of SNP marker effects (Aguilar *et al.* 2010).

The performances of the different GS methods have been tested for different livestock species and traits (Hayes *et al.* 2010; Colombani *et al.* 2013; Chen *et al.* 2014; Neves *et al.* 2014). The best method in terms of accuracy will depend on some factors, such as the number of phenotyped animals, heritability, effective population size, size of the genome, marker density, and genetic architecture of the trait (Daetwyler *et al.* 2008; Goddard 2009; Meuwissen 2009). In general, Bayesian methods outperform the GBLUP method for traits that are affected by a few large QTL, whereas for traits that are affected by many QTL with small effects, GBLUP would likely perform better than or similar to the Bayesian methods (Chen *et al.* 2014). Furthermore, Hayes *et al.* (2010) suggested that results obtained from cattle may not be relevant for other species, owing to the larger linkage disequilibrium (LD) blocks in bovine than other species.

Therefore, it is valuable to compare the accuracies of different GS methodologies to identify the method that will result in the highest accuracy for the genetic evaluation of resistance to one of the most important bacterial diseases affecting sea rearing of rainbow trout, which in turn is one of the most widely distributed aquaculture species in the world. In addition, GS can be implemented using a cost-effective individual genotyping strategy using low-density panels without much loss of information (Cleveland and Hickey 2013). Recent empirical studies have demonstrated that low-density panels are sufficient to get higher accuracy using genomic EBV (GEBV) than EBV obtained from pedigree-based BLUP (PBLUP) for resistance against *P. salmonis* (Bangera *et al.* 2017) and sea lice (Tsai *et al.* 2016; Correa *et al.* 2017) in Atlantic salmon.

The objectives of this study were: (i) to compare the accuracy of EBV using PBLUP with that using GBLUP, ssGBLUP, Bayes C, and LASSO; and (ii) to test the accuracy of genomic prediction and PBLUP using different marker densities (0.5, 3, 10, 20, and 27 K) for resistance against *P. salmonis* in rainbow trout.

## Materials and Methods

### Challenge test and phenotypes

The rainbow trout (*O. mykiss)* used in this study were obtained from the breeding nucleus of Aguas Claras S.A. (Puerto Montt, Chile) and were challenge-tested for resistance against *P. salmonis* at Aquainnovo’s Aquaculture Technology Center Patagonia, Puerto Montt, Chile (Flores-Mara *et al.* 2017). The fish used in this study were from the year-class 2011, which has undergone three generations of selection for growth, carcass quality, and appearance traits. Juveniles from 105 families (representing progeny from 105 dams and 48 sires) were reared in separate tanks until being individually tagged using a passive integrated transponder tag at an average weight of 7 g. After tagging, the animals were communally reared in a single tank for ∼7 months before being transferred to Aquainnovo’s research station (Lenca River, Xth Region, Chile). The fish were subjected to acclimation period during 20 d at the research station. After this period, a total of 2416 juveniles (with an average of 23 fish per family and ranging from 15 to 30 individuals) were experimentally challenged with *P. salmonis*. Before the challenge test, all fish were proven to be negative to the presence of infectious salmon anemia virus, infectious pancreatic necrosis virus, and *Renibacterium salmoninarum* by real-time PCR, and *Flavobacterium spp*. by culture. Fish were infected by injecting 0.2 ml of an LD50 (median lethal dose) inoculum of *P. salmonis* through intraperitoneal (IP) injection. Post IP injection, infected fish were equally distributed by family into three different tank replicates (used as fixed effect for PBLUP and genomics models). The challenge test continued for 32 d, and mortality and weight at the end of the experiment were recorded in all fish. All surviving fish at day 32 were anesthetized and killed. Tissue samples (fin clips) for genomic DNA isolation were taken from all dead and surviving fish and preserved in 95% ethanol at −80°.

Resistance to SRS was defined as the number of days to death (DD), with values ranging from 5 to 32; and binary survival (BS), scored as 1 if the fish died during the challenge test and 0 if the fish survived until the end of the challenge test.

### Genotypes

The genotyped individuals were selected to obtain a balanced number of animals per family (mean = 19, range from 12 to 26) and maintain the phenotypic variance. Genomic DNA was extracted from fin clip samples from 2130 fish (average of 19 fish per family, range from 12 to 26 fish) using a commercial DNeasy Blood & Tissue Kit, Qiagen, following the manufacturer’s instructions. The fish were genotyped using a commercially available 57 K Affymetrix Axiom SNP array, designed by the National Center for Cool and Cold Water Aquaculture at the United States Department of Agriculture (Palti *et al.* 2015).

The genotypes were subjected to quality control (QC) using Affymetrix’s Axiom Analysis Suite software, using the default settings (dish QC ≥ 0.82 and genotype call rate ≥ 97% for each sample). Additional QC steps were conducted by filtering out SNPs and samples with a Hardy–Weinberg equilibrium test *p*-value < 0.00001, SNP call rate lower than 0.90, and minor allele frequency lower than 0.01.

### Statistical models

#### Pedigree-based BLUP:

The pedigree-based variance components and EBV were estimated using BLUP and were compared with genomic evaluations. The model used was as follows:

y=Xβ+Zg+e,(M1)

where y is a vector of phenotypes (DD or BS), β is a vector of fixed effects (tank and body weight), g  is a vector of random additive polygenic genetic effects that follows a normal distribution ∼N(0, A$\sigma_{g}^{2}$), X and Z are incidence matrices, A is the additive relationship matrix, e is the random residual error with a distribution $∼\text{N}\left(0,I\sigma_{e}^{2}\right)$, and I is the identity matrix (Lynch and Walsh 1998). Body weight was included as a covariate in the analysis given that it significantly (*p* < 0.05) affected both traits. This was most likely because inoculum was IP-injected in the same dose for all fish, disregarding their initial size.

#### Genomic BLUP:

The SNP-based variance components and GEBV were estimated using GBLUP, in a similar way to the PBLUP model (M1), as implemented in the BLUPF90 software package (Misztal *et al.* 2016). The GBLUP model is a modification of the PBLUP method, where *g* is a vector of random additive genetic polygenic effects with a distribution ∼N$(0,G\sigma_{g}^{2})\;$and G is the genomic relationship matrix as described by VanRaden (2008). The G matrix is constructed based on all markers, and it can differ from the pedigree-based numerator relationship matrix (*A*), in that it can potentially have some negative off-diagonal values when individuals are molecularly less related than average pairs of animals in the sense of identity by state if the population were in Hardy–Weinberg equilibrium (Toro *et al.* 2002). The variance components, PBLUP, and GBLUP solutions for the breeding values were obtained using a restricted maximum likelihood method implemented in AIREMLF90, from the BLUPF90 family of programs (Misztal *et al.* 2016).

#### Single-step GBLUP:

The ssGBLUP model is similar to the PBLUP model (M1) except for the use of a combined genomic and pedigree relationship. The kinship matrix used was *H* (Aguilar *et al.* 2010), in which genotype and pedigree data are combined. The inverse of the matrix *H* is:

$$H^{-1}=A^{-1}+\left[\begin{array}{cc} 0 & 0\\ 0 & G^{-1}-A_{22}^{-1} \end{array}\right],$$ (1)

where $A^{-1}$ is the inverse numerator relationship matrix for all animals, $A_{22}^{-1}$ is the inverse of a pedigree-based relationship matrix for genotyped animals only, and $G^{-1}$ is the inverse genomic relationship matrix.

The EBV and the GEBV for DD were analyzed as linear traits using AIREMLF90 and BLUPF90. BS was analyzed using a threshold model (including a probit link function to transform event incidence to liability) by means of a Bayesian approach implemented in the THRGIBBS1F90 module from the BLUPF90 family of programs (Misztal *et al.* 2016). For Bayesian analysis (THRGIBBS1F90) 200,000 iterations were used in the Gibbs sampling, with a burn-in period of 20,000 iterations, and samples were saved every 50 cycles. Visual inspection of trace plots of the posterior variance components generated by POSTGIBBSF90 were used for QC purposes regarding convergence.

#### Bayes C:

Bayes C fits a mixture model that assumes some known fraction of markers have zero effects, and it has been shown that Bayes C is less sensitive to prior assumptions than, *e.g.*, Bayes B (Habier *et al.* 2011). All model parameters for Bayes C are defined as in M1, except the elements of vector g which was calculated for each fish as:

$$\sum_{i=1}^{n}g_{i}a_{i}\delta_{i},\left(\text{M2}\right)$$

where $g_{i}$ is the vector of the genotypes for the *i*th SNP for each animal; $a_{i}$ is the random allele substitution effect of the *i*th SNP; and δ_i_ is an indicator variable (0,1) sampled from a binomial distribution with parameters determined such that 1% of the markers were included in the model. The prior assumption is that SNP effects have independent and identical mixture distributions, where each marker has a point mass at zero with probability π and a univariate normal distribution with probability 1 − π having a null mean and variance $\sigma_{a}^{2}$, which in turn has a scaled inverse chi-squared prior, with $v_{a}=4$ and $v_{e}=10$ degrees of freedom (d.f.) and scale parameter $\sigma_{a}^{2}$ (or $\sigma_{e}^{2}$) (Fernando and Garrick 2013). For the additive variance, d.f. = 4 was used so the data would not overwhelm the prior if many loci were fitted, considering that, for Bayes C, a common locus variance is assumed and estimated by combining information from the prior and the data, and each fitted locus contributes to estimation of the common locus variance from the data (Fernando and Garrick 2013). The residual variance d.f. values were chosen based on those used in previous studies (Peters *et al.* 2012; Santana *et al.* 2016; Wolc *et al.* 2016; Yoshida *et al.* 2017).

#### Bayesian Lasso:

LASSO (Legarra *et al.* 2012) appears to be an interesting alternative method for performing regression on markers, suggesting that a double exponential prior may be a better choice than the Bayes A method, when most markers do not have an effect. The parameters for the LASSO method are defined as above in M1, except for an *a priori* distribution of individual SNP effects (*a_i_*) which was calculated as:

$$\mathrm{Pr}\left(a_{i}|\tau^{2}\right)N(1,\tau_{i}^{2})\;\mathrm{and}\;\mathrm{Pr}\left(a_{i}|\tau_{i}^{2}\right)=\frac{\lambda^{2}}{2}\exp(-\lambda^{2}|\tau_{i}^{2}),\left(\text{M3}\right)$$

where $\tau_{i}^{2}$ is the individual variance for each SNP, estimated conditionally on a regularization parameter λ (initial value was $\lambda^{2}=2/\sigma_{g}^{2}),$ which was estimated using an *a priori* gamma distribution bounded between 0 and 10^7^.

The Bayes C and LASSO analyses were performed using GS3 software (Legarra *et al.* 2012). A total of 200,000 iterations were used in the Gibbs sampling, with a burn-in period of 20,000 cycles where results were saved every 50 cycles. Convergence and autocorrelation were assessed by visual inspection of trace plots of the posterior variance components.

#### Genetic parameters and heritability:

The total additive genetic variance ($\sigma_{g}^{2}$) was estimated using relationship matrices *A*, *G*, and *H* for PBLUP, GBLUP, and ssGBLUP, respectively. For both DD and BS, the heritabilities were computed using the following equation:

$$h^{2}=\frac{\sigma_{a}^{2}}{\sigma_{a}^{2}+\sigma_{e}^{2}}.$$ (2)

For Bayesian models, the total additive genetic variance $\left(V_{A}^{\prime }\right)$ was estimated as the sum of the additive marker $\left(2\sigma_{a}^{2}\pi \sum^{\text{​}}p_{i}q_{i}\right)$ and the polygenic pedigree ($\sigma_{g}^{2}$)-based additive genetic variance $\left(V_{A}^{\prime }=2\sigma_{a}^{2}\pi \sum^{\text{​}}p_{i}q_{i}+\sigma_{g}^{2}\right)$, and the heritability was computed as:

$$h^{2}=\frac{V_{A}^{\prime }}{V_{A}^{\prime }+\sigma_{e}^{2}}.$$ (3)

#### Prediction accuracy:

The predictive abilities of different models were assessed using a fivefold cross-validation scheme. Briefly, all phenotyped and genotyped animals were randomly separated into five validation sets. The genomic predictions of the validation data sets were determined one at a time, where the phenotypic records of the validation fish (20% of the population) were set to missing and all remaining individuals with phenotypes and genotypes (80% of the population) were used as the training data set. For ssGBLUP, training and validation data sets were separated as described above, with the addition of 100% of the animals with only phenotypes (*n* = 482) into the training set.

Accuracy was used to assess the performance of each model for the validation set, and was estimated as:

$$r_{GEBV,BV}=\frac{r_{GEBV,y}}{h},$$ (4)

where $r_{GEBV,y}$ is the correlation between the EBV or GEBV of a given model (predicted for the validation set using information from the training set) and the record phenotype, and h is the square root of the pedigree-based estimate of heritability (Legarra *et al.* 2008; Ødegård *et al.* 2014).

In addition, the prediction accuracies obtained using different SNP densities were tested for all the methods. The 0.5 K, 3 K, 10 K, and 20 K SNP densities were randomly selected five times for each test method from the ∼27 K SNP that passed QC.

The bias of EBV prediction was obtained as the regression coefficient of phenotyped animals and EBV or GEBV, for PBLUP and genomics methods (GBLUP, ssGBLUP, Bayes C, and LASSO) in the validation data.

### Data availability

All phenotypic and genotypic data used in the current study can be found at the Figshare public repository (https://figshare.com/s/5219597a19f23873fda3).

## Results

### Descriptive statistics and genetic parameters

Summary statistics for both traits and covariates (body weight at the end of the challenge test) are presented in Table 1. The average DD ranged from 22 to 24 d and from 23 to 25 d between tanks for phenotyped (*n* = 2320) and genotyped (*n* = 1844) animals, respectively. The proportion of cumulative mortality ranged from 0.59 to 0.65 d and from 0.52 to 0.60 d between tanks for phenotyped and genotyped animals, respectively. The average body weights at the end of the challenge test were 165.3 g (SD = 40.44 g) and 168.8 g (SD = 41.37 g) for phenotyped and genotyped fish, respectively. A total of 1934 animals and 27,490 SNP (27 K) passed QC.

**Table 1:** Summary statistics for resistance against *Piscirickettsia salmonis* for phenotyped and genotyped rainbow trout

Traits	Tank	***N****^a^*	Mean	SD	Minimum	Maximum
Phenotyped fish						
Days to death (d)	1	819	23.59	8.07	5	32
** **	2	805	22.82	8.03	6	32
** **	3	792	22.13	8.27	7	32
Binary survival (1 or 0)	1	819	0.59	0.49	0	1
** **	2	805	0.65	0.48	0	1
** **	3	792	0.65	0.48	0	1
Final challenge weight (g)*^b^*	—	2320	165.30	40.44	46	295
Genotyped fish						
Days to death (d)	1	669	24.92	7.64	10	32
** **	2	641	24.01	7.78	11	32
** **	3	624	23.25	8.07	11	32
Binary survival (1 or 0)	1	669	0.52	0.50	0	1
** **	2	641	0.59	0.49	0	1
** **	3	624	0.60	0.49	0	1
Final challenge weight (g)*^b^*	—	1844	168.80	41.37	66	295

a Number of fish.

b Used as covariable.

Variance components estimates for all the models are presented in Table 2. For both DD and BS, the additive genetic variance and heritability were higher for genomic methods compared with PBLUP. For PBLUP the heritabilities were 0.38 and 0.54 for DD and BS, respectively. For genomic prediction methods the heritability values ranged from 0.45 to 0.57 and from 0.54 to 0.62 for DD and BS, respectively. For both traits, the lowest and the highest heritability estimates when using genomic prediction methods were obtained from the GBLUP and Bayes C methods, respectively.

**Table 2:** Estimates of residual variance ($\sigma_{e}^{2}$), total additive genetic variance ($V\prime_{a}$), and heritability (*h*^2^) for resistance against *Piscirickettsia salmonis* in rainbow trout

Methods	Traits							
	Days to death				Binary survival			
	$V_{a}^{\prime }$*^a^*	$\sigma_{e}^{2}$	h^2^	SE*^b^*	$V_{a}^{\prime }$*^a^*	$\sigma_{e}^{2}$	h^2^	SE*^b^*
PBLUP	23.017	37.375	0.381	0.059	1.177	1.005	0.539	0.053
LASSO	29.031	32.840	0.468	0.037	1.342	1.000	0.569	0.042
GBLUP	27.313	33.813	0.447	0.037	1.249	1.005	0.554	0.036
ssGBLUP	34.585	34.376	0.502	0.037	1.355	1.004	0.574	0.035
BAYES C	41.580	31.030	0.566	0.041	1.782	1.000	0.624	0.055

a Total additive genetic variance for PBLUP, ssGBLUP, and GBLUP was $\sigma_{g}^{2};$ for LASSO and BAYES C it was $2\sigma_{a}^{2}\pi \sum^{\text{​}}p_{i}q_{i}$+$\sigma_{p}^{2}$ ($\sigma_{p}^{2}=\;$polygenic effect).

b SE or SD for Bayesian methods.

### Accuracy of different methods and marker densities

Based on the fivefold cross-validation, the prediction accuracy of GEBV from genomic methods outperformed that of the EBV from PBLUP (Table 3). Within all genomic methods, the accuracies predicted for DD were higher than those for BS, with a low SE of the estimate (Table 3).

**Table 3:** Mean accuracy, bias, and SE of EBV and GEBV for resistance against *Piscirickettsia salmonis* using a 27 K SNP panel

Methods	Traits							
	Days to death				Binary survival			
	Accuracy	SE	Bias*^a^*	SE	Accuracy	SE	Bias*^a^*	SE
PBLUP	0.613	0.097	1.053	0.113	0.470	0.105	0.269	0.109
LASSO	0.784	0.069	0.968	0.069	0.591	0.090	0.253	0.041
GBLUP	0.785	0.064	1.026	0.092	0.598	0.082	0.240	0.049
ssGBLUP	0.798	0.061	1.035	0.091	0.608	0.082	0.267	0.048
BAYES C	0.859	0.061	1.063	0.102	0.614	0.086	0.240	0.045

a Regression for the EBV obtained by PBLUP and GEBV predicted with the different genomic methods.

**Figure 1:**
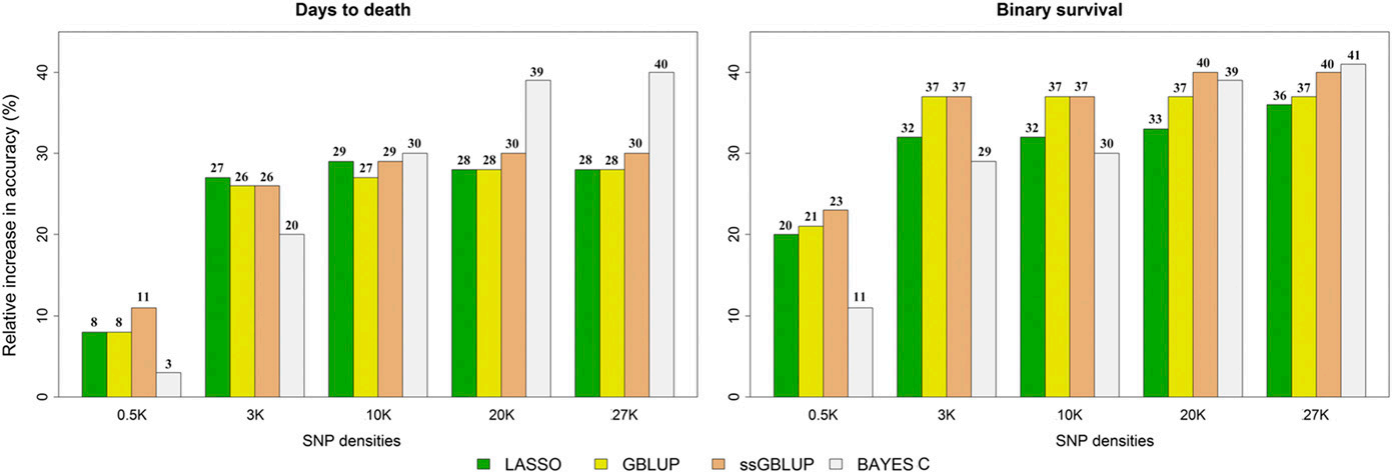
Relative increase in accuracy of different genomic selection methods for traits days to death and binary survival compared with PBLUP in rainbow trout using different SNP chip densities (0.5, 3, 10, 20, and 27 K).

The relative increase in accuracy of predicted GEBV compared with EBV from PBLUP varied moderately between models and traits at 27 K marker density (Figure 1). For both traits, the Bayes C method resulted in higher relative improvement in accuracy (>40%). On the other hand, LASSO and GBLUP resulted in the lowest relative increases in accuracy, and were the same (28%) for DD and similar for BS (LASSO = 36% and GBLUP = 37%) (Figure 1).

For marker density equal to 20 K, the Bayes C and ssGBLUP methods were most favorable in terms of relative increase in accuracy for DD and BS, respectively (Figure 1). At marker densities of 3 K and 10 K, ssGBLUP and GBLUP resulted in the same relative increase in accuracy for BS (Figure 1). By contrast, for DD the Bayesian methods had better performance. The ssGBLUP method performed slightly better than the other genomics methods at the lowest marker density (0.5 K), especially compared with Bayes C, which showed the lowest increase in accuracy for both traits (<11%). Nevertheless, the relative increases in accuracy of predicted GEBV from all genomic models were superior to those of EBV from PBLUP, even at the lowest marker density of 0.5 K for both traits. In general, the relative increase in accuracy was considerably more evident for BS than DD.

The GEBV estimated using GBLUP had the smallest departure from unity for DD. By contrast, the Bayes C method resulted in the most biased estimate (1.035). The bias values for EBV and GEBV for BS were considerably lower than 1.0 for all methods and ranged from 0.24 to 0.27, which indicates that all results for BS were upward biased (Table 3).

## Discussion

### Heritability for pedigree-based and genomic models

Low to moderate heritability estimates (from 0.16 to 0.24) have been reported for SRS resistance in Atlantic salmon (Yáñez *et al.* 2013, 2014) and coho salmon (Yáñez *et al.* 2016a) using a pedigree-based method to analyze a trait defined similarly to DD and BS. The comparatively higher estimates of heritability reported using genomic information compared with PBLUP in our study are in accordance with what has been reported in other fish species (Tsai *et al.* 2016; Bangera *et al.* 2017; Correa *et al.* 2017; Vallejo *et al.* 2017). Vallejo *et al.* (2016, 2017) also estimated a similar range of heritability using genomic models (0.26–0.54) and PBLUP (0.31–0.48) for bacterial cold water disease resistance in rainbow trout.

### Prediction accuracy

The relatively high accuracy achieved in the present study for genomic methods suggested that the strong relationship between the animals in the training and validation data sets, and the small effective population size of this breeding population, could contribute to the accuracy values. This in turn could result in extensive LD and a smaller number of effective chromosome segments to be estimated. The GEBV prediction accuracy for resistance against cold water disease in rainbow trout was estimated using different methods by Vallejo *et al.* (2017), and the accuracies reported were similar of magnitude for survival days (0.63–0.71) and survival status (0.66–0.71).

In Atlantic salmon, Bangera *et al.* (2017) and Correa *et al.* (2017) showed that the relative increase in GEBV prediction accuracies from different models compared with PBLUP was up to 30 and 22% higher for resistance against SRS and *Caligus rogercresseyi*, respectively. However, improvement in accuracy values in the current study varied from 28 to 41%; this was still lower than the values reported by Vallejo *et al.* (2017), which ranged from 83 to 109% for bacterial cold water disease resistance in rainbow trout. We speculate that in the study of Vallejo *et al.* (2017), the use of a larger number of animals with phenotype in the training data set (7893 *vs.* 2417) resulted in a higher relative increase in accuracy. Furthermore, Piyasatian *et al.* (2007) suggested that high heritability of a trait (>0.45 in the present study) reduced the benefit of GS over PBLUP.

### Effect of marker density on accuracy

Genotyping of large numbers of selection candidates with high-density panels may not be cost-effective if the economic benefit per animal is low compared with the cost of genotyping (Habier *et al.* 2009), as in aquaculture species. The use of low-density panels, with considerable reduction in cost of genotyping, is a potential cost-effective approach to implement GS. Previous studies in Atlantic salmon reported that low-density panels between 5 and 10 K were sufficient to obtain reliable increases in accuracy (even close to the maximal accuracy of high-density panels) compared with PBLUP (Tsai *et al.* 2015, 2016; Correa *et al.* 2017). The lowest-density SNP panel (0.5 K) used in our study resulted in the lowest accuracies, mainly for DD, as a result of insufficient LD between the markers owing to the large distance between the randomly selected low-density markers (Bangera *et al.* 2017).

We suggest that the considerable gain in GEBV accuracy obtained in different genomic prediction methods using markers above 10 K was because of the high LD between the randomly selected markers. All low-density panels showed improved GEBV accuracy over PBLUP (Figure 1); higher accuracy of genomic prediction can be obtained by using high-density panels, as also shown by Ødegård *et al.* (2014) and Bangera *et al.* (2017). Therefore, to implement cost-effective GS, a strategy of genotyping of the selection candidates with a low-density panel (*e.g.*, 500 SNPs) followed by imputation to a high-density panel (*e.g.*, 50 K) could be used (Tsai *et al.* 2017). Imputing from 0.25 to 0.5 K to a high-density panel and using the imputed genotypes for genomic prediction was shown to achieve a similar level of accuracy compared with using true genotypes in Atlantic salmon (Tsai *et al.* 2017).

### Comparison of models at different marker densities

The GBLUP approach assumes polygenic control of the trait and makes use of all genotyped SNPs for calculating the genomic relationship matrix. By contrast, Bayesian models assume that a few markers explain the genetic variance of a trait (Habier *et al.* 2007; Hayes *et al.* 2009; de los Campos *et al.* 2013). Thus, Bayesian methods are expected to perform better than GBLUP when several moderate- to large-effect QTL are controlling the trait. In this study, two GBLUP and two Bayesian methods were tested to compare the accuracy of genomic predictions from different GS models with those obtained by ordinary PBLUP.

All genomic prediction methods outperformed PBLUP at different SNP densities (Figure 1). For both traits, the Bayes C method had the highest accuracy (>40% relative increase over PBLUP) at the highest SNP density (27 K). The GBLUP and ssGBLUP methods showed a constant relative increase in accuracy from 3 K to 27 K SNP panels, mainly for BS. Interestingly, for the 0.5 K SNP panel, ssGBLUP resulted in the highest accuracies for both traits, suggesting that for very low-density panels, the use of additional animals with only phenotypes in the training set can improve the accuracy of predictions. Furthermore, ssGBLUP could be used as a strategy to reduce the genotyping costs and still achieve higher GEBV accuracies compared with PBLUP. As has been reported previously, the use of information from genotyped and nongenotyped individuals (Lourenco *et al.* 2014) and the increase in accuracy when compared with PBLUP (Chen *et al.* 2011; Christensen *et al.* 2012) are among the advantages of using ssGBLUP.

The use of progressively more markers in the GBLUP method might have resulted in better capturing of genetic relationships, whereas Bayes C was more effective in capturing LD between markers and QTL when more markers were used (Bermingham *et al.* 2015). Furthermore, fitting 1% of the SNPs with larger effect in the Bayes C method resulted in the highest relative increase in accuracy. This is most likely owing to the genetic architecture of *P. salmonis* resistance in rainbow trout. In a previous GWAS in the same population, *P. salmonis* resistance was suggested to be under oligogenic control (data not published), with a few SNPs showing moderate to large effects (the top 10 SNPs explained >50% of the genetic variance; results not shown).

Bayes C outperformed ssGBLUP at 20 and 27 K SNP densities for DD, and had slightly lower performance for BS. However, for lower SNP densities, the Bayes C method had lower accuracy (Figure 1 and Table 3). The large distance between the low-density SNPs results in lower LD between the markers and QTL. The possibility of exclusion of the SNPs with moderate to high effects during the process of random selection might have resulted in lower relative accuracies in Bayes C.

Several other studies also reported that GEBV estimated by Bayesian methods outperformed EBV estimated using pedigree-based methods, and even other genomic methods (*i.e.* GBLUP and ssGBLUP) (Neves *et al.* 2014; Vallejo *et al.* 2016, 2017; Bangera *et al.* 2017; Correa *et al.* 2017). A disadvantage in using Bayesian methodologies (*e.g.*, Bayes C) is the considerably higher computational time, which could increase linearly depending on the number of markers fitted in the model (Bermingham *et al.* 2015). Considering the similarity in accuracies between Bayes C and ssGBLUP, and the highest accuracies for the low-density panels (Figure 1 and Table 3), the ssGBLUP method may be a more flexible and computationally efficient alternative.

### Bias

GEBV bias was calculated as the regression of EBV on GEBV. A regression coefficient equal to one is indicative of predictions that are on a scale similar to that of the GEBV, whereas a regression <1 or >1 indicates that GEBV is overestimated or underestimated, respectively. Here, we found bias values somewhat below unity for BS, indicating that GEBV was underregressed compared with EBV, and suggesting that the genetic trends could be underestimated and have negative impact in selection schemes. Other studies reported bias values <1 for BS, and similar bias values to those of the present work for DD (Vallejo *et al.* 2016, 2017; Bangera *et al.* 2017).

### Implications

Our results showed that using genomic information for estimating breeding values achieved higher accuracies compared with using only pedigree information for both DD and BS. Using 20 K and 3 K SNP panels for DD and BS, respectively, was enough to improve accuracy to similar values to those obtained for 27 K SNP chip density. Given the economic importance of resistance against *P. salmonis* in rainbow trout, and the efficacy of genomic prediction over pedigree-based methods, we suggest that selective breeding using genomic information will be an important component to control SRS and reduce losses in aquaculture systems.
